# Viewing the immune checkpoint VISTA: landscape and outcomes across cancers

**DOI:** 10.1016/j.esmoop.2024.102942

**Published:** 2024-03-18

**Authors:** D. Nishizaki, R. Kurzrock, H. Miyashita, J.J. Adashek, S. Lee, M. Nikanjam, R.N. Eskander, H. Patel, G.P. Botta, M.K. Nesline, S. Pabla, J.M. Conroy, P. DePietro, J.K. Sicklick, S. Kato

**Affiliations:** 1 Center for Personalized Cancer Therapy and Division of Hematology and Oncology, Department of Medicine, University of California San Diego, Moores Cancer Center, La Jolla; 2 MCW Cancer Center and Genomic Sciences and Precision Medicine Center, Medical College of Wisconsin, Milwaukee, USA; 3 WIN Consortium, Paris, France; 4 Dartmouth Cancer Center, Hematology and Medical Oncology, Lebanon; 5 Department of Oncology, The Sidney Kimmel Comprehensive Cancer Center, The Johns Hopkins Hospital, Baltimore; 6 Center for Personalized Cancer Therapy and Division of Gynecologic Oncology, Department of Obstetrics, Gynecology, and Reproductive Sciences, University of California San Diego, Moores Cancer Center, La Jolla; 7 OmniSeq Inc., Buffalo; 8 Division of Surgical Oncology, Department of Surgery, Center for Personalized Cancer Therapy, University of California San Diego, La Jolla, USA

**Keywords:** precision medicine, gene expression profiling, neoplasms, immunotherapy, immune checkpoint inhibitors

## Abstract

**Background:**

Optimizing immune checkpoint inhibitor (ICI) therapy may require identification of co-targetable checkpoint pathways via immune profiling. Herein, we analyzed the transcriptomic expression and clinical correlates of V-domain immunoglobulin suppressor of T-cell activation (VISTA), a promising targetable checkpoint.

**Patients and methods:**

RNA sequencing was carried out on 514 tissues reflecting diverse advanced/metastatic cancers. Expression of eight immune checkpoint markers [lymphocyte-activation gene 3 (LAG-3), tumor necrosis factor receptor superfamily 14 (TNFRSF14), programmed cell death protein 1 (PD-1), programmed death-ligand 1 (PD-L1), programmed death-ligand 2 (PD-L2), B- and T-lymphocyte attenuator (BTLA), T-cell immunoglobulin and mucin domain-containing protein 3 (TIM-3), cytotoxic T-lymphocyte antigen 4 (CTLA-4)], in addition to VISTA, was analyzed, along with clinical outcomes.

**Results:**

High VISTA RNA expression was observed in 32% of tumors (66/514) and was the most common highly expressed checkpoint among the nine assessed. High VISTA expression was independently correlated with high BTLA, TIM-3, and TNFRSF14, and with a diagnosis of pancreatic, small intestine, and stomach cancer. VISTA transcript levels did not correlate with overall survival (OS) from metastatic/advanced disease in the pan-cancer cohort or with immunotherapy outcome (progression-free survival and OS from the start of ICI) in 217 ICI-treated patients. However, in ICI-treated pancreatic cancer patients (*n* = 16), median OS was significantly shorter (from immunotherapy initiation) for the high- versus not-high-VISTA groups (0.28 versus 1.21 years) (*P* = 0.047); in contrast, VISTA levels were not correlated with OS in 36 pancreatic cancer patients who did not receive ICI.

**Conclusion:**

High VISTA expression correlates with high BTLA, TIM-3, and TNFRSF14 checkpoint-related molecules and with poorer post-immunotherapy survival in pancreatic cancer, consistent with prior literature indicating that VISTA is prominently expressed on CD68+ macrophages in pancreatic cancers and requiring validation in larger prospective studies. Immunomic analysis may be important for individualized precision immunotherapy.

## Highlights

• VISTA expression differs among cancer types and there are diverse expression patterns.
• High VISTA transcript correlates with high BTLA, TIM-3, and TNFRSF14 expression.
• High VISTA expression may predict poor immunotherapy survival in pancreatic cancer patients.
• Co-targeting VISTA and co-expressed immunoregulatory molecules merit further investigation.
• Tailored combined immunotherapy with immune profiling warrants prospective studies.

## Introduction

The emergence of immune checkpoint inhibitors (ICIs) has drastically changed cancer therapy. Negative checkpoint regulators, including cytotoxic T-lymphocyte antigen 4 (CTLA-4), programmed cell death protein 1 (PD-1), programmed death-ligand 1 (PD-L1), and lymphocyte-activation gene 3 (LAG-3), have been successfully targeted in several cancers.^1^, ^2^, ^3^, ^4^ However, existing checkpoint inhibitors targeting these negative regulators are effective in only a small portion of patients and even among responders, some relapse.^5^ Based on the underlying mechanisms of resistance, and as it is recognized that multiple checkpoints and their pathways regulate anticancer immune responsiveness,^6^ additional immune checkpoint molecules that can be co-targeted or targeted after relapse have been investigated to overcome this resistance.

V-domain immunoglobulin suppressor of T-cell activation (VISTA), first reported in 2011,^7^^,^^8^ is an immune checkpoint protein that bears homology to PD-L1 and is primarily expressed on hematopoietic cells.^9^ Expression of VISTA on antigen-presenting cells suppresses T-cell proliferation and its cytokine production.^7^ VISTA regulates T-cell receptor activation and controls peripheral tolerance under normal conditions.^10^ Also, VISTA regulates innate immunity that leads to inflammatory responses.^11^ Unlike other checkpoint inhibitors known so far, VISTA is expressed on naïve T cells and contributes to quiescence of T cells as the earliest checkpoint.^12^

**Figure 1:**
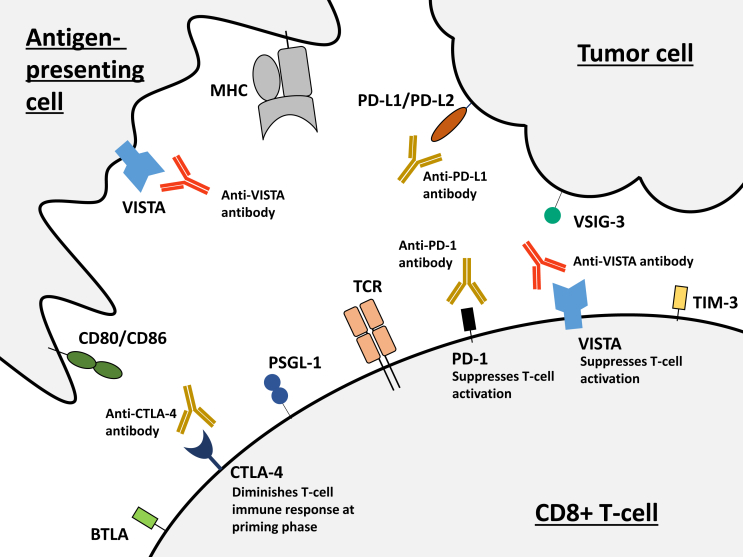
**Putative mechanism of action of VISTA and interaction with currently targetable immune checkpoints.** Antigen-presenting cells and T cells express VISTA, which can combine with VSIG-3 on tumor cells^42^^,^^43^ and PSGL-1 on T cells.^31^^,^^44^^,^^45^ Blocking VISTA may promote T-cell activation (several anti-VISTA antibodies are currently under investigation; see Supplementary Table S1, available at https://doi.org/10.1016/j.esmoop.2024.102942). CTLA-4 and PD-1 expressed on T cells bind to CD80/CD86 on antigen-presenting cells and PD-L1/PD-L2 on tumor cells, respectively, reducing T cells’ antitumor activity. CTLA-4, PD-1, and PD-L1 are currently targetable; targeting them in combination with VISTA blockade may bring better outcomes. Other checkpoints, including TIM-3 and BTLA, which were highly co-expressed with VISTA in the current study (see Table 1 and Figure 3), are expressed on T cells.^46^ These checkpoints may also be potentially targetable. BTLA, B- and T-lymphocyte attenuator; CTLA-4, cytotoxic T-lymphocyte antigen 4; MHC, major histocompatibility complex; PD-L1, programmed death-ligand 1; PD-L2, programmed death-ligand 2; PSGL-1, P-selectin glycoprotein ligand 1; TCR, T-cell response; TIM-3, T-cell immunoglobulin and mucin domain-containing protein 3; VISTA, V-domain immunoglobulin suppressor of T-cell activation; VSIG-3, V-Set and immunoglobulin domain containing 3.

Within the tumor microenvironment, VISTA is expressed at high levels on myeloid cells and Foxp3+CD4+ regulatory T cells.^9^^,^^13^ In mice tumor models, overexpression of VISTA diminishes the antitumor immune response, and VISTA blockade using a monoclonal antibody enhances T-cell responses *in vitro* and *in vivo*.^7^ Anti-VISTA monoclonal antibodies predominantly regulate infiltration of effector T cells and alter the tumor microenvironment.^13^ Clinically, VISTA expression in the tumor microenvironment has been reported to be an independent prognostic factor for worse survival in patients with melanoma.^14^ Our previous study using the transcriptome has shown that high VISTA expression was associated with shorter progression-free survival (PFS) after anti-PD-1/PD-L1-based therapies in the pan-cancer setting.^15^ Moreover, VISTA has a non-redundant role that is different from the PD-1/PD-L1 pathway in regulating T-cell activation.^16^ Further, VISTA expression levels increased in patients with melanoma and prostate cancer, respectively, previously treated by PD-1 or CTLA-4 inhibitors.^17^^,^^18^ VISTA has also been implicated as a potential target in pancreatic cancer since it is overexpressed on macrophages in the stromal area of pancreatic cancer compared to melanoma.^19^ Taken together, VISTA might be targetable in combination with other checkpoint inhibitors including PD-1 and CTLA-4 inhibitors (Figure 1).^6^ Currently, several clinical trials evaluating the safety of anti-VISTA agents are underway (Supplementary Table S1, available at https://doi.org/10.1016/j.esmoop.2024.102942).^20^, ^21^, ^22^, ^23^ In these trials, anti-VISTA drugs are administered solely or in combination with pembrolizumab.

Herein, we investigated the RNA expression of VISTA and its correlation with various immune markers and other immunoregulatory molecules in order to better understand the potentially actionable immune environment.

## Patients and methods

### Patients

This was an observational study on personalized medicine. The cohort included 514 patients who were treated for diverse solid cancers at the University of California, San Diego Moores Cancer Center for Personalized Therapy. The current study evaluated patients with a confirmed metastatic or unresectable cancer diagnosis, regardless of age, sex, or cancer type, and availability of a tumor tissue specimen, which was suitable for subsequent immunomic analysis. The RNA expression levels of 395 immune markers, including nine checkpoints, were examined at OmniSeq (https://www.omniseq.com/), a Clinical Laboratory Improvement Amendments (CLIA)-licensed and College of American Pathologist (CAP)-accredited clinical laboratory. If a patient had >1 sample collected on different days, we used the sample from an earlier time point for analysis. We curated the clinical information, including the patients’ age, sex, cancer type, microsatellite instability (MSI) status, tumor mutation burden (TMB), and PD-L1 immunohistochemistry (IHC), from the electronic medical records. All investigations were conducted in accordance with the guidelines of the Institutional Review Board of University of California San Diego for data collection (Study of Personalized Cancer Therapy to Determine Response and Toxicity, UCSD_PREDICT, NCT02478931), and any investigational interventions for which patients or their legal guardians provided written informed consent.

### Tissue collection and immune expression analysis

Formalin-fixed, paraffin-embedded (FFPE) samples were obtained and evaluated using RNA sequencing at OmniSeq laboratory after collection. Total RNA was extracted using the truXTRAC FFPE extraction kit (Covaris, Inc., Woburn, MA), with some modifications to the manufacturer’s instructions. RNA was dissolved in 50 μl of water, and the yield was measured through Quant-iT RNA HS assay (Thermo Fisher Scientific, Waltham, MA), following the manufacturer’s recommendations.

The predetermined titer of 10 ng RNA was set as the acceptance criterion for appropriate library preparation. Torrent Suite’s plugin immuneResponseRNA (v5.2.0.0) was used to generate absolute reads, and the RNA expression levels of 395 different genes were measured.^24^ Among them, we focused on nine genes that are related to immune checkpoints: VISTA, PD-1, PD-L1, programmed death-ligand 2 (PD-L2), T-cell immunoglobulin and mucin domain-containing protein 3 (TIM-3), LAG-3, B- and T-lymphocyte attenuator (BTLA), CTLA-4, and tumor necrosis factor receptor superfamily 14 (TNFRSF14) (Supplementary Table S2, available at https://doi.org/10.1016/j.esmoop.2024.102942). To allow next generation sequencing (NGS) measurements across runs to be comparable for evaluation and interpretation, background-subtracted read counts were subsequently normalized into normalized reads per million (nRPM) values.^24^ These normalized expression values (nRPM) were further ranked from 0 to 100 against a reference population of 735 previously tested pan-cancer solid tumor samples, spanning 35 different tumor histologies, various stages, age groups, and genders. This reference population represents a real-world, clinically tested population with a wide dynamic range of immune expression in solid tumors that were predominantly pre-treated. Ranking against this population allowed us to classify high expression of specific immune response genes. A threshold of 75th for high expression represents the top quartile of expression seen in the reference population; therefore, this is an arbitrary yet reasonable threshold.^25^ Subsequently, the expression profiles of checkpoint markers were stratified into ‘high’ (75-100 percentile), ‘moderate’ (25-74 percentile), and ‘low’ (0-24 percentile) with their rank values.

### Variable definition

To measure PD-L1 expression level, three different IHC assays were used: Dako PD-L1 22C3 pharmDx assay, Dako PD-L1 28-8 pharmDx assay (Dako North America, Inc., Carpinteria, CA, *n* = 475 and *n* = 6, respectively), and VENTANA PD-L1 SP142 assay (Ventana Medical Systems, Inc., Tucson, AZ, *n* = 33). PD-L1 IHC was based on clinical practice. PD-L1 expression with IHC was deemed positive if the combined positive score was ≥1% using 22C3, the score of tumor-infiltrating immune cells (ICs) was ≥1% with SP142, or the score of tumor cells (TCs) was ≥1% using 28-8.

To calculate TMB, genomic DNA was extracted from qualified FFPE tumors (>30% neoplastic nuclei) by means of the truXTRAC FFPE extraction kit (Covaris) with 10 ng of DNA input for library preparation. DNA libraries were prepared with Ion AmpliSeq targeted sequencing chemistry using the Comprehensive Cancer Panel, followed by enrichment and template preparation using the Ion Chef system, and sequencing on the Ion S5XL 540 chip (Thermo Fisher Scientific). After the removal of germline variants, synonymous variants, indels, and single nucleotide variants with <5% variant allele frequency, TMB was reported as suitable mutations per appropriate panel size (mutations/megabase).

For assessing MSI, the truXTRAC FFPE extraction kit (Covaris) was used to retrieve genomic DNA from qualified FFPE tumors (>20% neoplastic nuclei). The MSI-NGS assay examined 29 homopolymer loci, including BAT-25 and BAT-26, by sequencing tumor DNA (20 ng) on an Illumina MiSeq Sequencer (Illumina, Inc., San Diego, CA).^26^ MSI-NGS Caller, a computational tool of the assay, compares the tumor homopolymer repeat profile of a sample to a normal allele distribution predefined at each locus, and accordingly makes MSI calls (unstable, stable, or inconclusive) without the need for a matched normal DNA.

### Endpoints and statistical analyses

Patient characteristics and the expression patterns of immune checkpoint markers were summarized using descriptive statistics. To investigate the association between high RNA expression of VISTA, various immune markers, and cancer types, we carried out univariable logistic regression with cancer types that had 25 or more samples and/or a higher proportion of VISTA-high patients than average and all checkpoint markers. Subsequent multivariable logistic regression was carried out with variables that had *P* values ≤0.05 in the univariable analysis.

Survival analyses were carried out for patients with survival information using the Kaplan–Meier method. Overall survival (OS) was defined as the duration from the date of metastatic or locally advanced disease to the date of the last follow-up.

To determine the effect of immunotherapy on outcomes, Kaplan–Meier analysis was limited to patients who underwent immunotherapy. OS was defined as the duration from the initial date of immunotherapy to the date of last follow-up, and PFS was defined as the duration from the initial date of immunotherapy to the date of the earliest of disease progression (clinical or radiological) or death from any cause. Patient survival was stratified by the level of VISTA expression, namely high (≥75th percentile rank) versus moderate/low (<75th percentile rank), and was compared using the log-rank test. Subgroup survival analysis was *post hoc* based on the multivariable logistic regression and the subgroup number of patients. The data cut-off date for the current analysis was 24 June 2022. All statistical analyses were carried out using R 4.2.1 (R Foundation for Statistical Computing, Vienna, Austria). A *P* value of ≤0.05 was deemed statistically significant.

## Results

### Patient characteristics

Among the 514 patients in the cohort, 310 (60%) were women, and the median age was 61 years (range 24-93 years). The most frequent cancer type was colorectal cancer [27% (140/514)], followed by pancreatic cancer [11% (55/514)], breast cancer [9.5% (49/514)], ovarian cancer [8.4% (43/514)], and stomach cancer [4.9% (25/514)] (Supplementary Figure S1 and Table S3, available at https://doi.org/10.1016/j.esmoop.2024.102942). Out of the 514 patients, 489 had confirmed metastatic or locally advanced disease; however, for 25 patients, the dates of their metastatic disease remained undocumented. Overall, 217 patients received ICIs; 199 received anti-PD-1/PD-L1 alone, 2 received anti-CTLA-4 alone, and 16 received simultaneous anti-PD-1/PD-L1 and anti-CTLA-4. The other 272 patients never received immunotherapy.

### VISTA transcript levels were high in almost one-third of cancers

**Figure 2:**
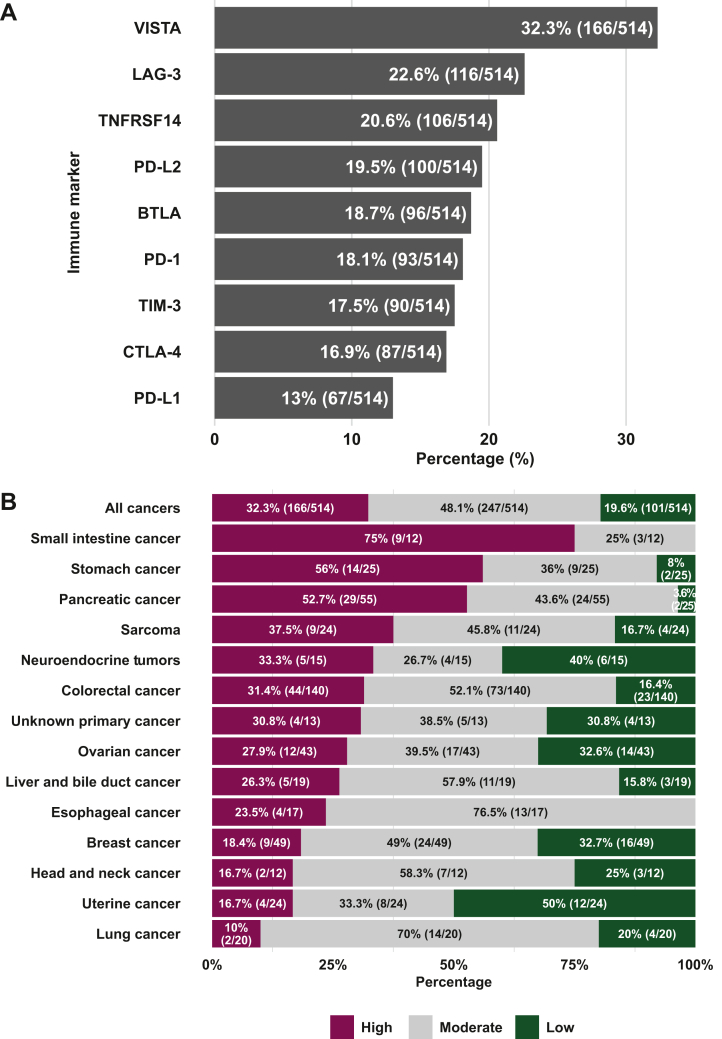
**Proportion of patients with high VISTA expression.** (A) Proportion of patients with high RNA expression (≥75th percentile rank compared to controls) among nine checkpoint markers. The most frequent high expression marker was VISTA, which showed high expression in 166 of 514 patients (32.3%). (B) Proportion of patients with high, moderate, and low RNA expression of VISTA stratified by cancer type. High RNA expression of VISTA was observed in small intestine cancer [75% (9/12)], followed by stomach cancer [56% (14/25)], pancreatic cancer [52.7% (29/55)], sarcoma [37.5% (9/24)], and neuroendocrine tumors [33.3% (5/15)]; all of which were more than the average proportion (32.3%) across all cancers. Definition of RNA expression: high = 75-100, moderate = 25-74, and low = 0-24 percentile rank value score (see Patients and methods). BTLA, B- and T-lymphocyte attenuator; CTLA-4, cytotoxic T-lymphocyte antigen 4; LAG-3, lymphocyte-activation gene 3; PD-1, programmed cell death protein 1; PD-L1, programmed death-ligand 1; PD-L2, programmed death-ligand 2; TIM-3, T-cell immunoglobulin and mucin domain-containing protein 3; TNFRSF14, tumor necrosis factor receptor superfamily 14; VISTA, V-domain immunoglobulin suppressor of T-cell activation.

VISTA expression was high (percentile rank value: 75-100) in 32% (166/514) of patients, moderate (percentile rank value: 25-74) in 48% (247/514), and low (percentile rank value: 0-24) in 20% (101/514). VISTA was the most frequent highly expressed marker among nine checkpoint markers assessed, followed by LAG-3 [22.6% (116/514)], TNFRSF14 [20.6% (106/514)], PD-L2 [19.5% (100/514)], BTLA [18.7% (96/514)], PD-1 [18.1% (93/514)], TIM-3 [17.5% (90/514)], CTLA-4 [16.9% (87/514)], and PD-L1 [13.0% (67/514)] (Figure 2A).

### High VISTA transcripts independently correlated with a diagnosis of pancreatic, small intestine, and stomach cancer and were negatively associated with breast cancer

Stratified by cancer type, high RNA expression of VISTA was most commonly observed in small intestine cancer [75% (9/12)], followed by stomach cancer [56% (14/25)], pancreatic cancer [52.7% (29/55)], sarcoma [37.5% (9/24)], and neuroendocrine tumors [33.3% (5/15)] [all listed above had more than the average proportion among all cancer types (32.3%)]. Conversely, colorectal cancer [31.4% (44/140)], unknown primary cancer [30.8% (4/13)], ovarian cancer [27.9% (12/43)], liver and bile duct cancer [26.3% (5/19)], esophageal cancer [23.5% (4/17)], breast cancer [18.4% (9/49)], uterine cancer [16.7% (4/24)], head and neck cancer [16.7% (2/12)], and lung cancer [10% (2/20)] had less proportion than the average (Figure 2B).

In multivariate analysis, high RNA expression of VISTA was positively associated with small intestine cancer [odds ratio (OR) 5.87, 95% confidence interval (CI) 1.48-29.3, *P* = 0.017], stomach cancer (OR 3.05, 95% CI 1.23-7.61, *P* = 0.015), and pancreatic cancer (OR 1.95, 95% CI 1.00-3.77, *P* = 0.048), and negatively associated with breast cancer (OR 0.32, 95% CI 0.12-0.75, *P* = 0.013) (Table 1).

**Table 1:** Clinical and immune characteristics associated with high VISTA expression (≥75th percentile RNA rank) (*n* = 514)

Characteristics			Univariable		Multivariable	
Name	Condition	Proportion of high VISTA expression	Odds ratio (95% CI)	*P* value	Odds ratio (95% CI)	*P* value
Age (years)	≥61	33% (85/256)	1.09 (0.75-1.57)	0.661		
	<61	31% (81/258)	—			
Sex	Male	33% (68/204)	1.08 (0.74-1.58)	0.683		
	Female	32% (98/310)	—			
MSI^a^	High	47% (7/15)	1.95 (0.67-5.53)	0.205		
	Not high	31% (144/465)	—			
TMB (mutations/Mb)^b^	≥10	21% (7/33)	0.67 (0.26-1.52)	0.370		
	<10	29% (119/417)	—			
PD-L1 IHC	Positive^c^	30% (47/156)	0.86 (0.57-1.29)	0.476		
	Negative	33% (119/357)	—			
PD-1	High	54% (50/93)	3.06 (1.93-4.86)	<0.0001	1.03 (0.51-2.04)	0.923
	Low/moderate	28% (116/421)	—		—	
PD-L1	High	37% (25/67)	1.29 (0.75-2.19)	0.347		
	Low/moderate	32% (141/447)	—			
PD-L2	High	56% (56/100)	3.52 (2.25-5.54)	<0.0001	1.77 (0.96-3.22)	0.063
	Low/moderate	27% (110/414)	—		—	
CTLA-4	High	59% (51/87)	3.84 (2.39-6.23)	<0.0001	1.35 (0.68-2.65)	0.391
	Low/moderate	27% (115/427)	—		—	
BTLA	High	61% (59/96)	4.63 (2.92-7.44)	<0.0001	2.35 (1.26-4.39)	**0.007**
	Low/moderate	26% (107/418)	—		—	(associated with higher level of VISTA)
LAG-3	High	47% (54/116)	2.22 (1.45-3.40)	<0.0001	1.26 (0.70-2.21)	0.428
	Low/moderate	28% (112/398)	—		—	
TIM-3	High	64% (58/90)	5.30 (3.29-8.68)	<0.0001	3.15 (1.73-5.79)	**0.0002**
	Low/moderate	25% (108/424)	—		—	(associated with higher level of VISTA)
TNFRSF14	High	57% (60/106)	3.72 (2.39-5.81)	<0.0001	2.74 (1.65-4.54)	**0.0001**
	Low/moderate	26% (106/408)	—		—	(associated with higher level of VISTA)
Colorectal cancer	Yes	31% (44/140)	0.95 (0.62-1.43)	0.797		
	No	33% (122/374)	—			
Pancreatic cancer	Yes	53% (29/55)	2.62 (1.49-4.64)	0.001	1.95 (1.00-3.77)	**0.048**
	No	30% (137/459)	—		—	(associated with higher level of VISTA)
Breast cancer	Yes	18% (9/49)	0.44 (0.20-0.89)	0.032	0.32 (0.12-0.75)	**0.013**
	No	34% (157/465)	—		—	(associated with lower level of VISTA)
Ovarian cancer	Yes	28% (12/43)	0.80 (0.38-1.56)	0.521		
	No	33% (154/471)	—			
Stomach cancer	Yes	56% (14/25)	2.82 (1.25-6.50)	0.012	3.05 (1.23-7.61)	**0.015**
	No	31% (152/489)	—		—	(Associated with higher level of VISTA)
Neuroendocrine tumor	Yes	33% (5/15)	1.05 (0.32-3.01)	0.931		
	No	32% (161/499)	—			
Small intestine cancer	Yes	75% (9/12)	6.59 (1.94-30.0)	0.005	5.87 (1.48-29.3)	**0.017**
	No	31% (157/502)	—		—	(associated with higher level of VISTA)
Sarcoma	Yes	38% (9/24)	1.27 (0.52-2.92)	0.577		
	No	32% (157/490)	—			

Variables with *P* ≤ 0.05 from univariate analyses were included for multivariate analysis. Bold values indicate *P* ≤ 0.05.

BTLA, B- and T-lymphocyte attenuator; CI, confidence interval; CTLA-4, cytotoxic T-lymphocyte antigen 4; IHC, immunohistochemistry; LAG-3, lymphocyte-activation gene 3; Mb, megabase; MSI, microsatellite instability; PD-L1, programmed death-ligand 1; PD-L2, programmed death-ligand 2; TIM-3, T-cell immunoglobulin and mucin domain-containing protein 3; TMB, tumor mutation burden; TNFRSF14, tumor necrosis factor receptor superfamily 14; VISTA, V-domain immunoglobulin suppressor of T-cell activation.

a MSI was available in 480 patients.

b Among 514 patients, TMB was available in 450 patients.

c PD-L1 was deemed positive if the combined positive score (CPS) was ≥1% or the score of tumor-infiltrating immune cells (ICs) was ≥1%.

### Patterns of highly expressed immune markers along with VISTA were heterogeneous across cancers

**Figure 3:**
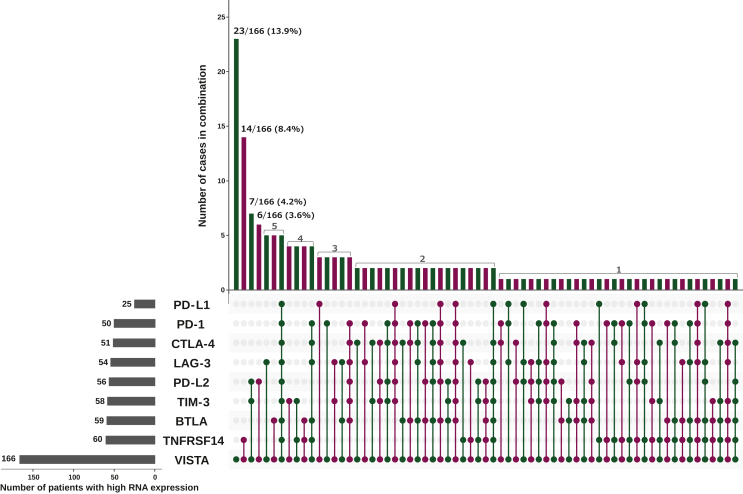
**The UpSet plot depicts combination pattern of high expression of VISTA (≥75thpercentile rank compared to controls) and that of other checkpoint markers among patients who had high VISTA expression (*n* = 166).** This plot visualizes the simultaneous RNA expression pattern as a matrix, in which the rows represent the immune markers and the columns represent their overlaps. The bars on the rows show the number of patients with high RNA expression; the bars on the columns show the number of patients who had the same combination pattern of high RNA expression. The bars on the columns and the corresponding dots and bars in the matrix are alternately colored to facilitate readability. High VISTA expression was most frequently accompanied by high TNFRSF14 (*n* = 60), followed by high BTLA (*n* = 59), and high TIM-3 (*n* = 58; left axis). The most prevalent expression pattern was high VISTA expression only [23/166 (13.9%)], followed by co-expression of high VISTA and high TNFRSF14 [14/166 (8.4%)], and simultaneous expression of high VISTA, TIM-3, and PD-L2 [7/166 (4.2%)]. BTLA, B- and T-lymphocyte attenuator; LAG-3, lymphocyte-activation gene 3; PD-1, programmed cell death protein 1; PD-L1, programmed death-ligand 1; PD-L2, programmed death-ligand 2; TIM-3, T-cell immunoglobulin and mucin domain-containing protein 3; TNFRSF14, tumor necrosis factor receptor superfamily 14; VISTA, V-domain immunoglobulin suppressor of T-cell activation.

Figure 3 shows the combination pattern of high RNA expression among 166 patients with high VISTA transcript levels. Heterogeneous combination patterns were observed. The most common pattern was high expression of VISTA only [13.9% (23/166)], followed by co-high expression with TNFRSF14 [8.4% (14/166)], co-high expression with TIM-3 and PD-L2 [4.2% (7/166)], and co-high expression with PD-L2 [3.6% (6/166)]. Throughout all patterns, VISTA was accompanied by high expression levels of TNFRSF14 (*n* = 60), BTLA (*n* = 59), TIM-3 (*n* = 58), PD-L2 (*n* = 56), LAG-3 (*n* = 54), CTLA-4 (*n* = 51), PD-1 (*n* = 51), and PD-L1 (*n* = 25).

### High VISTA expression independently correlated with high BTLA, TIM-3, and TNFRS14

The results of the univariable and subsequent multivariable logistic regression are shown in Table 1. In multivariate analysis, among immune markers, high RNA expression of VISTA was significantly associated with that of BTLA (OR 2.35, 95% CI 1.26-4.39, *P* = 0.007), TIM-3 (OR 3.15, 95% CI 1.73-5.79, *P* = 0.0002), and TNFRSF14 (OR 2.74, 95% CI 1.65-4.54, *P* = 0.0001).

### VISTA transcript levels did not have a prognostic impact in pan-cancer survival analysis

**Figure 4:**
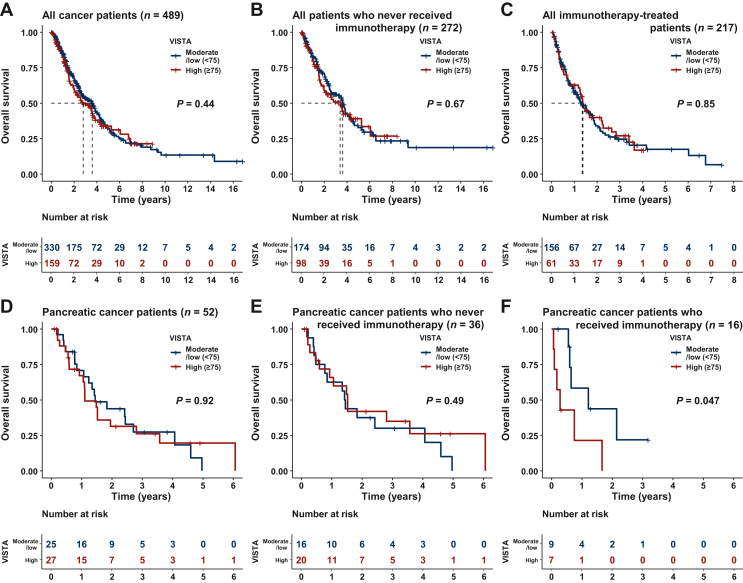
**Survival outcomes stratified by VISTA expression levels.** OS for all cancer patients (*n* = 489) (A), and all cancer patients who never received immunotherapy (*n* = 272) (B), with high (≥75th percentile rank) versus medium/low VISTA levels; OS was assessed from the date of metastatic/advanced disease. VISTA level did not have a prognostic association with OS. VISTA levels also did not have a predictive impact on immunotherapy OS across cancers (C), with OS assessed from the start of first immunotherapy-containing regimen. Patients with pancreatic cancer were also analyzed because pancreatic cancer was associated with high VISTA levels (see Table 1) in multivariable analysis and because it was a group of patients with *n* > 50. High versus medium/low VISTA RNA levels did not have a prognostic association with OS from metastatic/advanced disease in pancreatic cancer (D and E). However, in patients with pancreatic cancer who received immunotherapy, high VISTA levels were predictive of significantly shorter OS from the start of immunotherapy (F). Survival was evaluated in 489 patients because the date of metastatic disease was not documented in 25 patients. Definition of RNA expression: high = 75-100, moderate = 25-74, and low = 0-24 percentile rank value score (see Patients and methods). (A) Among all patients, median OS times from the date of metastatic or locally advanced disease was 2.81 years (95% CI 2.12-3.82 years) for the high-VISTA group and 3.61 years (95% CI 2.71-4.07 years) for the low-VISTA group, respectively (*P* = 0.44). (B) Among patients who did not receive immunotherapy, median survival times from the date of metastatic or locally advanced disease was 3.41 years (95% CI 1.97 years-NE) for the high-VISTA group and 3.63 years (95% CI 2.59-4.25 years) for the low-VISTA group, respectively (*P* = 0.67). (C) Among all 217 immunotherapy-treated patients, median OS after the initiation of immunotherapy was 1.38 years (95% CI 1.18-2.27 years) for the high-VISTA group and 1.35 years (95% CI 1.07-1.82 years) for the low-VISTA group, respectively (*P* = 0.85). (D) Pancreatic cancer patients (*n* = 52) had a median OS of 1.11 years for the high-VISTA group and 1.45 years for the low-VISTA group, respectively (*P* = 0.92), from metastatic/advanced disease. (E) Pancreatic cancer patients who never received immunotherapy (*n* = 36) did not show significant difference in median OS from time of metastatic/advanced disease between the high- and low-VISTA groups [1.52 years (95% CI 0.77 years-NE) versus 1.43 years (95% CI 0.93 years-NE)]. (F) In pancreatic cancer patients who underwent immunotherapy (*n* = 16), median OS was significantly lower from start of immunotherapy (0.28 years) for the high-VISTA group versus 1.21 years for the low-VISTA group (*P* = 0.047). CI, confidence interval; NE, not estimable; OS, overall survival; VISTA, V-domain immunoglobulin suppressor of T-cell activation.

Survival outcomes were evaluable among 489 patients with confirmed metastatic or locally advanced disease, and the median follow-up period was 2.04 years (interquartile range 1.05-3.63 years); among them, 272 patients never received immunotherapy and 217 patients were treated with an immunotherapy-based regimen (Supplementary Figure S1, available at https://doi.org/10.1016/j.esmoop.2024.102942, Figure 4). Among the 489 patients, median OS from the date of metastatic or locally advanced disease to last follow-up was 2.81 years (95% CI 2.12-3.82 years) for the high-VISTA group and 3.61 years (95% CI 2.71-4.07 years) for the low-VISTA group, respectively (*P* = 0.44; Figure 4A). Regarding patients who never received immunotherapy (Supplementary Table S4, available at https://doi.org/10.1016/j.esmoop.2024.102942), the median OS from the date of metastatic or locally advanced disease to last follow-up was 3.41 years [95% CI 1.97 years-not estimable (NE)] for the high-VISTA group and 3.63 years (95% CI 2.59-4.25 years) for the low-VISTA group, respectively (*P* = 0.67; Figure 4B), showing no impact of VISTA levels.

### VISTA transcript levels were not associated with PFS or OS across cancers after immunotherapy

VISTA levels did not have a predictive correlation with OS after immunotherapy across cancers (Figure 4C) (with OS assessed from the start of the first immunotherapy-containing regimen). Among all 217 immunotherapy-treated patients (Supplementary Table S5, available at https://doi.org/10.1016/j.esmoop.2024.102942), median OS after the initiation of immunotherapy was 1.38 years (95% CI 1.18-2.27 years) for the high-VISTA group and 1.35 years (95% CI 1.07-1.82 years) for the low-VISTA group, respectively, showing no impact of VISTA levels (*P* = 0.85).

Regarding PFS across cancers after immunotherapy (from the date of start of first immunotherapy) (Supplementary Figure S2, available at https://doi.org/10.1016/j.esmoop.2024.102942) based on VISTA levels, median PFS after the initiation of immunotherapy (*n* = 217) was 0.42 years (95% CI 0.31-0.86 years) for the high-VISTA group and 0.39 years (95% CI 0.33-0.48 years) for the low-VISTA group, respectively (*P* = 0.48; Supplementary Figure S2A, available at https://doi.org/10.1016/j.esmoop.2024.102942).

### High VISTA levels may correlate with poorer outcome in pancreatic cancer patients who received immunotherapy

Patients with pancreatic cancer were selected for disease-specific subgroup analysis because pancreatic cancer was significantly associated with high VISTA in multivariate analysis (Table 1), and pancreatic cancer represented >10% of the patients studied (Supplementary Table S3, available at https://doi.org/10.1016/j.esmoop.2024.102942).

Among pancreatic cancer patients (*n* = 52), median OS from the date of advanced/metastatic disease to last follow-up was 1.11 years for the high-VISTA group and 1.45 years for the low-VISTA group, respectively (*P* = 0.92, Figure 4D), indicating no prognostic impact of VISTA levels. Pancreatic cancer patients who never received immunotherapy also did not show a significant difference in median OS time between the high- and low-VISTA groups [1.52 years (95% CI 0.77 years-NE) versus 1.43 years (95% CI 0.93 years-NE); Figure 4E].

In contrast, in pancreatic cancer patients who underwent immunotherapy (*n* = 16), median OS was significantly lower from the start of immunotherapy (0.28 years) for the high-VISTA group versus 1.21 years for the low-VISTA group (*P* = 0.047) (Figure 4F). Pancreatic cancer patients who underwent immunotherapy (*n* = 16) had a median PFS of 0.14 years for the high-VISTA group and 0.64 years for the low-VISTA group, respectively (*P* = 0.12) (Supplementary Figure S2, available at https://doi.org/10.1016/j.esmoop.2024.102942), showing a trend toward shorter PFS in the high-VISTA group, with the small number of patients precluding definitive conclusions.

## Discussion

We analyzed the transcriptomic expression of the VISTA checkpoint in a pan-cancer setting. High VISTA expression was observed in 32% (166/514) of the patients. Notably, the prevalence of high VISTA expression varied across cancer types. Furthermore, most tumors [86% (143/166)] with high VISTA expression displayed complex co-expression patterns with other immune markers. High VISTA expression was independently correlated with high BTLA, TIM-3, and TNFRS14 checkpoints and with a diagnosis of pancreatic, small intestine, and stomach cancer. A significantly lower proportion of patients with breast cancer had high VISTA.

Across cancers, regarding the outcome analyses, VISTA levels did not function as a predictive factor following immunotherapy or as a prognostic factor after the development of advanced metastatic disease. Even so, among patients with pancreatic cancer treated with immunotherapy, those with high VISTA expression exhibited significantly worse survival compared to those with moderate or low VISTA expression (median OS 0.28 versus 1.21 years, *P* = 0.047). This finding suggests that treatment outcomes after VISTA-targeted therapy may not be uniform across different primary cancer sites. Indeed, VISTA has been reported to be a potential target in pancreatic cancer, as VISTA is overexpressed predominantly on macrophages in the stromal area of pancreatic cancer compared to melanoma.^19^ Moreover, we previously reported that 63% of pancreatic ductal adenocarcinoma patients showed high VISTA expression,^27^ which is consistent with the current findings of ∼53% of pancreatic tumors having high VISTA. To date, effective anti-PD-1/PD-L1 immunotherapy for pancreatic cancer has been limited to patients with mismatch repair deficiency and high TMB, representing only 1%-2% of patients with pancreatic cancer.^28^^,^^29^ Thus, new therapeutic options are needed. It is plausible that up-regulation of alternative checkpoints, such as VISTA, in pancreatic and other cancers serves as a resistance factor, and that incorporation of immune profiling to identify patients who may benefit from specific immune modulators merits investigation.

Because VISTA is frequently co-expressed with other checkpoints, a combination approach to immunotherapy might be necessary. Indeed, VISTA, PD-1/PD-L1, and CTLA-4 play different roles at different stages of the cancer–immunity cycle,^12^^,^^30^ suggesting a rationale for the combination of immunomodulators. To date, several *in vivo* studies have shown the effectiveness of VISTA-targeted drugs in combination with PD-1/PD-L1 or CTLA-4 inhibitors, although their effectiveness varies among drug combinations and cancer types.^16^^,^^30^^,^^31^ Although rational combination immunotherapy is a promising approach, the interaction of VISTA with other immune checkpoints may differ depending on the cancer type and expression patterns or magnitude of immune modulators, which are complex and diverse, as demonstrated in the present study.

The complexity of the immune co-expression patterns illustrated in the current study recapitulates the genomic complexity of cancer. Thus, lessons learned from genomics-based single-agent and combination therapy^32^ may be applicable to immunotherapy. Selecting tumors based on their molecular makeup is the *sine qua non* of effective gene-directed therapy^33^, ^34^, ^35^, ^36^ and may be critical for immunotherapy as well; customized combination approaches based on patient immunomics may be essential to improve outcomes. Current trials in immunotherapy and daily practice generally assess the IHC of PD-L1, microsatellite status, and TMB as biomarkers of responsiveness; however, a more intensive investigation of the immune milieu may be important. RNA sequencing can quantitatively evaluate the expression patterns of diverse immune mediators that may vary across cancer types and even within the same tumor type.^37^ In this context, RNA sequencing from tissue specimens could be a powerful tool to discern the immune landscape of an individual tumor for optimal treatment selection.

The prognostic impact of VISTA has not been clearcut.^38^, ^39^, ^40^ Our current study suggests that there is no difference in the prognosis (survival from advanced/metastatic disease) in 272 patients with high versus not-high tumor tissue VISTA in the pan-cancer setting (with these patients never being exposed to immunotherapy). In our previous report using RNA sequencing in a pan-cancer setting, high VISTA expression was associated with worse clinical outcomes among advanced cancer patients (*n* = 39) treated with checkpoint inhibitors.^15^ Meanwhile, in the present study, VISTA levels did not demonstrate a predictive impact following immunotherapy among patients with high VISTA expression (*n* = 217 patients). Notably, however, we observed that pancreatic cancer patients with high versus not-high RNA VISTA expression exhibited significantly worse survival when the patients were treated with immunotherapy (though VISTA RNA expression had no survival impact in the pancreatic patients who never received immunotherapy); the number of patients in the pancreatic cancer cohort is however small. Similar to our study, a prior report by Hou et al. found that high VISTA expression (IHC) occurred in a significant subset of pancreatic cancers; however, in contrast to our findings, high VISTA IHC in TCs (but not ICs or endothelial cells) was associated with better survival in patients with pancreatic cancer (immunotherapy treatment status not defined).^41^ This discrepancy could be attributed to the different technologies used to evaluate VISTA expression (IHC versus transcriptomics) or the criteria for defining ‘high’ VISTA expression. Further studies on the VISTA prognostic and immunotherapy predictive effect in pancreatic cancer are warranted.

There are several limitations in this study. Firstly, only a small number of patients with pancreatic cancer were analyzed. Although pancreatic cancer patients with high VISTA levels had shorter survival after immunotherapy than those with lower VISTA levels, implying that VISTA may be a resistance factor that needs to be co-targeted, our observations need to be validated in larger prospective cohorts. Secondly, the cut-off value used to declare high expression in RNA sequencing [≥75th percentile rank (compared to a reference population of 735 patients with diverse cancers)] may require adjustment. Thirdly, we carried out a cross-sectional analysis of immune markers, which can fluctuate throughout the disease course. Fourthly, we did not examine expression of VISTA by cell type; future studies should address this issue. Finally, larger cohorts of specific tumor types may need to be analyzed in future studies.

### Conclusions

In summary, VISTA expression differs among cancer types and its co-expression patterns are diverse. Most patients with high VISTA expression (86%) had at least one highly co-expressed immune marker. High VISTA might be an adverse predictive factor for immunotherapy responsiveness among patients with pancreatic cancer, a finding that, if validated in larger cohorts, suggests that co-targeting VISTA in these patients may warrant investigation. Our findings suggest that a customized combination immunotherapy approach based on individualized immune profiling merits further investigation to address immunomic heterogeneity between patients and to overcome immunotherapy resistance.
