# Supplementary material for: Viewing the immune checkpoint VISTA: landscape and outcomes across cancers
Source: ESMO Open. 2024 Mar 18;9(4):102942. doi: 10.1016/j.esmoop.2024.102942 (PMC10966162; doi:10.1016/j.esmoop.2024.102942)
Supplement: Supplementary data [file mmc1.pdf]

Supplemental File

Viewing the immune checkpoint VISTA: Landscape and outcomes across cancers

Daisuke Nishizaki[1#], Razelle Kurzrock[2,3#], Hirotaka Miyashita[4], Jacob J. Adashek[5], Suzanna Lee[1], Mina Nikanjam[1], Ramez N. Eskander[6], Hitendra Patel[1], Gregory P. Botta[1], Mary K. Nesline[7], Sarabjot Pabla[7], Jeffrey M. Conroy[7], Paul DePietro[7], Jason K. Sicklick[8], Shumei Kato[1]
# Equally contributed

1. Center for Personalized Cancer Therapy and Division of Hematology and Oncology, Department of Medicine, University of California San Diego, Moores Cancer Center, La Jolla, CA, United States.
2. MCW Cancer Center and Genomic Sciences and Precision Medicine Center, Medical College of Wisconsin, Milwaukee, WI, United States.
3. WIN consortium, Paris, France
4. Dartmouth Cancer Center, Hematology and Medical Oncology, Lebanon, NH, United States.
5. Department of Oncology, The Sidney Kimmel Comprehensive Cancer Center, The Johns Hopkins Hospital, Baltimore, MD, United States.
6. Center for Personalized Cancer Therapy and Division of Gynecologic Oncology, Department of Obstetrics, Gynecology, and Reproductive Sciences, University of California San Diego, Moores Cancer Center, La Jolla, CA, United States.
7. OmniSeq Inc., Buffalo, NY, United States.
8. Division of Surgical Oncology, Department of Surgery, Center for Personalized Cancer Therapy, University of California San Diego, La Jolla, CA United States.

Corresponding author:

Shumei Kato, M.D.
Center for Personalized Cancer Therapy and Division of Hematology and Oncology, Department of Medicine, University of California San Diego, Moores Cancer Center
3855 Health Science Drive, La Jolla, CA, 92037
Email: smkato@health.ucsd.edu
Phone: 858-822-2372
FAX: 858-822-6186

Daisuke Nishizaki, M.D.
Center for Personalized Cancer Therapy and Division of Hematology and Oncology, Department of Medicine, University of California San Diego, Moores Cancer Center
3855 Health Science Drive, La Jolla, CA, 92037
Email: dnishizaki@health.ucsd.edu
Phone: 858-822-2372
FAX: 858-822-6186

Supplemental Table 1. Selected clinical trials targeting VISTA

ID	NCT Number	Drug	Mechanism of Action	Combination	Planned Enrollment	Phases	Status	Conditions	Primary Outcome Measures	Was VISTA expression required for enrollment?
1	NCT 02671955	JNJ-61610588	Human IgG1 kappa anti-VISTA monoclonal antibody	None	12 participants	1	Terminated †	Advanced Cancer	Frequency of dose- limiting toxicity	Not required, but change from baseline in protein expression of VISTA was measured
2	NCT 04475523	CI-8993	Human IgG1 kappa anti-VISTA monoclonal antibody	None	50 participants	1	Active, not recruiting	Solid Tumor (non-lymphoma)	To determine the maximum tolerated dose of CI-8993	Not required
3	NCT 02812875	CA-170	Small molecule antagonist for PD-L1, PD-L2 and VISTA	None	71 participants	1	Completed	Advanced Solid Tumors or Lymphomas	The number of patients with a dose-limiting toxicity in the first treatment cycle	Not required
4	NCT 05082610	HMBD-002	Anti-VISTA monoclonal antibody	Pembrolizumab	240 participants	1	Recruiting	Advanced Solid Tumor	Dose-limiting Toxicity	Not required
5	NCT 04564417	W0180	Anti-VISTA monoclonal antibody	Pembrolizumab	69 participants	1	Recruiting	Locally Advanced or Metastatic Solid Tumors	Dose-limiting Toxicity	Not required
6	NCT 05708950	KVA 12123	Anti-VISTA fully human IgG1 antibody	Monotherapy or with pembrolizumab	314 participants	1/2	Recruiting	Advanced solid tumors	Adverse events through study completion	Not required
7	NCT 05864144	SNS-101	Anti-VISTA IgG1 monoclonal antibody	Monotherapy or with cemiplimab	129 participants	1/2	Recruiting	Advanced solid tumors	Adverse events; Determining the phase 2 dose	Not required

Last searched on 8/31/2023 at ClinicalTrials.gov and Cochrane Central Register of Controlled Trials.

† Company business decision. The license of JNJ-61610588 was transferred in early 2020 and the compound was designated as CI-8993. This means JNJ-61610588 is the same drug as CI-8993.

Supplemental Table 2. Function of each immune marker

Immune markers	Aliases	(Putative) Ligands and function in cancer immunity	Example of available and possible related immune modulators	References
PD-1 (Programmed Death 1)	CD279	PD-L1 and PD-L2 are expressed on the surface of tumor cells or antigen presenting cells, whereas PD-1 is expressed on T cells.<br><br>Binding PD-L1/PD-L2 to PD-1 triggers an inhibitory signal that suppresses anti-cancer immunity.<br><br>The Blockade of PD-1/PD-L1/PD-L2 axis activates anticancer immunity. Overexpression of PD-L1 is a predictive biomarker of benefit from the inhibition of the axis.	<u>Anti-PD-1-antibody</u><br>Nivolumab<br>Pembrolizumab<br>Cemiplimab<br>Dostarlimab<br><br><u>Anti-PD-L1-antibody</u><br>Atezolizumab<br>Avelumab<br>Durvalumab	S1
PD-L1 (Programmed Death Ligand 1)	CD274, B7-H1			S2
PD-L2 (Programmed Death Ligand 2)	B7-DC, PDCD1LG2, CD273			S3
CTLA-4 (Cytotoxic T-lymphocyte-associated protein 4)	CD152	CTLA-4 is expressed on regulatory T cells. CD80 (B7-1)/CD86 (B7-2), ligands of CTLA-4, are expressed on antigen presenting cells.<br><br>CTLA-4 has stronger affinity to CD80/CD86 than CD28, which binds to CD80/CD86 and then activates T cells. When CTLA-4 is bound to CD80/CD86, T cells are inactivated.	Ipilimumab<br>Tremelimumab<br>Bostensilimab *<br>BMS-986218 *<br>ONC-392 *	S4
BTLA (B and T lymphocyte attenuator)	CD272	The binding of TNFRSF14 to BTLA leads to immune inhibitor signal. Other TNFRSF14 ligands include CD160 and LIGHT.	Icatolimab *	S5
TNFRSF14 (Tumor Necrosis Factor Receptor Superfamily 14)	HVEM, CD270			

TIM-3 (T-cell immunoglobulin and mucin-domain containing-3)	HAVCR2, CD366	TIM-3 is expressed on CD8+ T cells, Tregs, NK cells, and dendric cells. Its ligands include GAL-9, CEACAM1, PtdSer, and HMGB1. TIM-3 expression is associated with T cell exhaution.	TSR-022 *<br>Sym023 *<br>LY3321367 *<br>RO7121661 *<br>LB1410 *	S6
LAG-3 (Lymphocyte Activation Gene 3)	CD223	LAG-3 is expressed on activated cytotoxic T cells and regulatory T cells. MHC is one of ligands of LAG-3 and others include LSECtin, GAL-3 and FGL1. LAG-3 and its ligands interaction suppresses T cell function.	Relatlimab<br>Tebotelimab *<br>RO7247669 *<br>Sym022 *	S7
VISTA (V-domain Ig Suppressor of T cell Activation)	B7-H5, Dies1, PD-1H	The ligands of VISTA include PSGL-1 and VSIG-3. PSGL-1 binds to VISTA in an acidic environment. The binding of VISTA to PSGL-1/VSIG-3 leads to inhibitory signals to anticancer immunity.	CI-8993 *<br>CA-170 (small molecule antagonist for PD-L1, PD-L2, and VISTA) *<br>HMBD-002 *<br>W0180 *<br>KVA12123 *	S8

Supplemental Table 3. Patient Characteristics (N = 514)

**Characteristic**	**N = 514**
Age, median (range, years)	61 (24, 93)
Gender, n (%)	
Female	310 (60%) **
Male	204 (40%)
PD-L1 IHC positive, n (%)* †	156 (30%)
TMB ≥10 (mutations/Megabase), n (%) *	33 (7%)
MSI unstable, n (%) *	15 (3%)
Disease, n (%)	
Colorectal Cancer	140 (27%)
Pancreatic Cancer	55 (11%)
Breast Cancer	49 (9.5%)
Ovarian Cancer	43 (8.4%)
Stomach Cancer	25 (4.9%)
Sarcoma	24 (4.7%)
Uterine Cancer	24 (4.7%) ***
Lung Cancer	20 (3.9%)
Liver and Bile Duct Cancer	19 (3.7%)
Esophageal Cancer	17 (3.3%)
Neuroendocrine Tumors	15 (2.9%)
Unknown Primary Cancer	13 (2.5%)
Head and Neck Cancer	12 (2.3%)
Small Intestine Cancer	12 (2.3%)
Other ‡	46 (8.9%)
Evaluable patients treated with immunotherapy	217 (42%)
Anti-PD-1/PD-L1 agents	199 (39%)
Anti-CTLA-4 monotherapy	2 (0.4%)
Anti-CTLA-4 and anti-PD-1/PD-L1 combination therapy	16 (3.1%)
Patients who received immunotherapy as a first line therapy	54 (11%)

Abbreviations: IHC, immunohistochemistry; MSI, microsatellite instability; TMB, tumor mutation burden.
* Tabulation was performed only among patients with available TMB (n = 450), MSI (n = 480), and PD-L1 IHC (n = 513).
** One patient whose gender was previously unknown was now verified to be male.
*** One patient was initially coded as female genital tract cancer; on review, the tumor was found to be uterine cancer and hence the total number of uterine cancers in this manuscript is 24.
† PD-L1 expression with IHC was deemed positive if the combined positive score (CPS) was ≥1% using 22C3, the score of tumor-infiltrating immune cells (IC) was ≥1% with SP142, or the score of tumor cells (TC) was ≥1% using 28-8.
‡ Cancers that had at least 10 patients were listed. Other includes Melanoma (n = 6), Cervical Cancer (n = 5), Bladder Cancer (n = 4), Gallbladder and Extrahepatic Bile Duct Cancers (n = 4), Prostate Cancer (n = 4), Brain and Nervous System Cancer (n = 3), Kidney and Renal Pelvis Cancer (n = 3), Squamous Cell Carcinoma of the Skin (n = 3), Thyroid Cancer (n = 3), Adrenal Gland Cancer (n = 2), Lipomatous Neoplasms (n = 2), Mesothelioma (n = 2), adrenal cortical carcinoma (n = 1), Basal Cell Carcinoma of the Skin (n = 1), ocular melanoma (n = 1), primary peritoneal carcinoma (n = 1), and Thymic cancer (n = 1).

Supplemental Table 4. Age, sex, and basic immune markers for overall survival analysis among patients who never received immunotherapy (N=272 patients were available for clinical follow-up).

Variable	VISTA High (≥75 percentile RNA rank) (N = 98)	VISTA Moderate/Low (<75 percentile RNA rank) (N = 174)	p-value *
Age (years), median (IQR)	60 (52, 69)	60 (49, 68)	0.23
Female, n (%)	64 (65%)	107 (61%)	0.60
PD-L1 IHC CPS ≥1, n (%)	20 (20%)	37 (21%)	0.88
MSI unstable, n (%)	2 (2.3%)	2 (1.2%)	0.61
TMB ≥10 (mutations/Megabase), n (%)	3 (3.9%)	6 (3.7%)	0.99
Cancer types			
Colorectal cancer	27 (28%)	52 (30%)	0.78
Pancreatic cancer	20 (20%)	16 (9.2%)	0.014
Breast cancer	4 (4.1%)	20 (11%)	0.045
Ovarian cancer	7 (7.1%)	17 (9.8%)	0.51
Stomach cancer	8 (8.2%)	13 (2.3%)	0.032

* Welch’s t-test or Fisher’s exact test was used.

Supplemental Table 5. Age, sex, and basic immune markers for overall survival analysis among patients who received immunotherapy at some point (N=217).

	VISTA		
Variable	**High** (≥75 percentile RNA rank) (N = 61)	**Moderate/Low** (<75 percentile RNA rank) (N = 156)	p-value *
Age (years), median (IQR)	62 (48, 71)	62 (52, 70)	0.80
Female, n (%)	30 (49%)	93 (59%)	0.22
PD-L1 IHC CPS ≥1, n (%)	25 (41%)	62 (39%)	0.88
MSI unstable, n (%)	5 (8.8%)	4 (2.7%)	0.12
TMB ≥10 (mutations/Megabase), n (%)	4 (9.1%)	18 (12%)	0.79
Cancer types			
Colorectal cancer	15 (25%)	39 (25%)	0.99
Pancreatic cancer	7 (11%)	9 (5.8%)	0.16
Breast cancer	4 (6.6%)	15 (9.6%)	0.60
Ovarian cancer	4 (6.6%)	14 (9.0%)	0.78
Stomach cancer	5 (8.2%)	6 (3.8%)	0.30

* Welch’s t-test or Fisher’s exact test was used.

Supplemental Figure 1. Patient flow diagram.

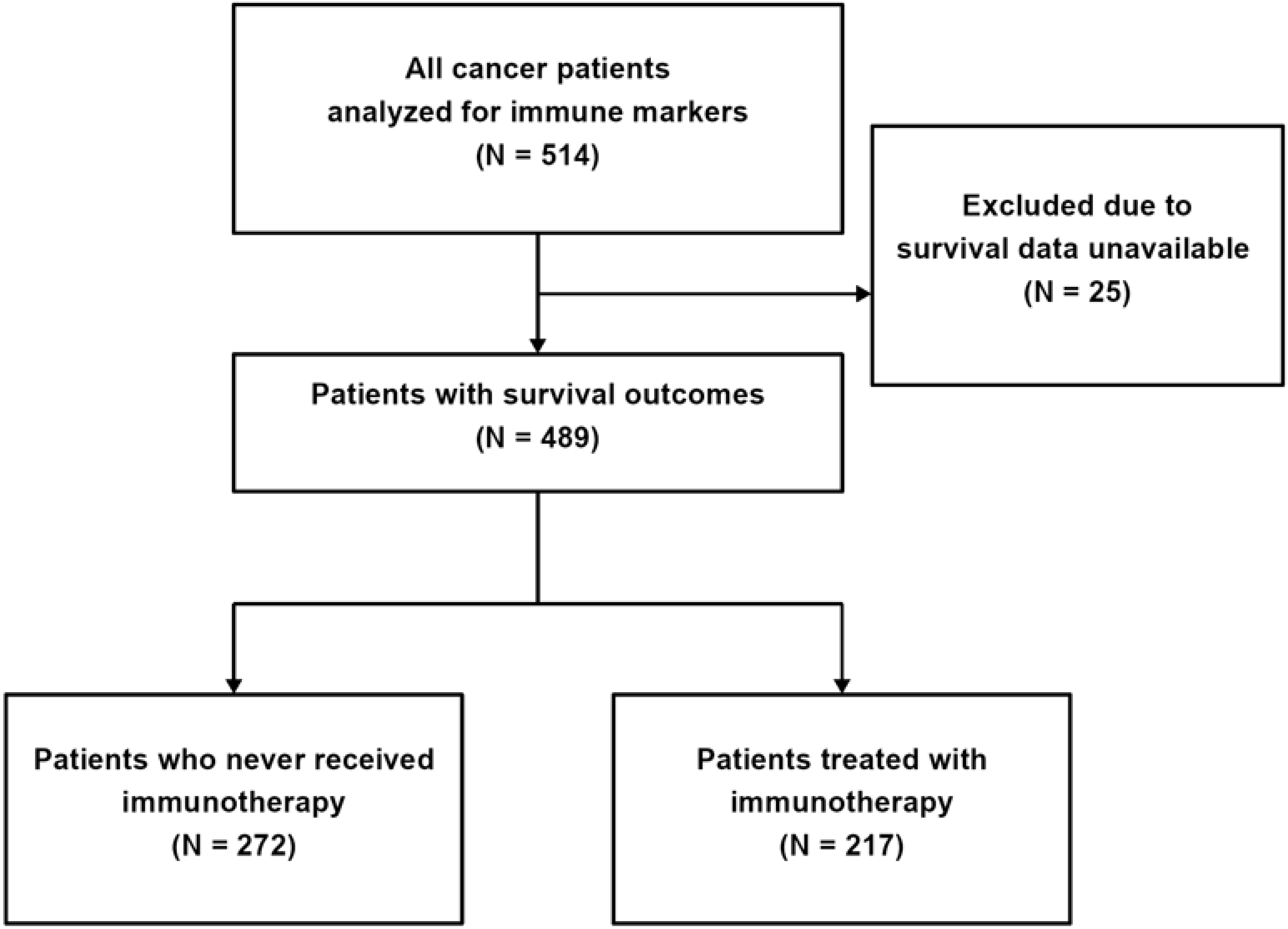

Supplemental Figure 2. Progression-free survival (from the start of first immunotherapy) for immunotherapy-treated patients (N=217) and their subgroups

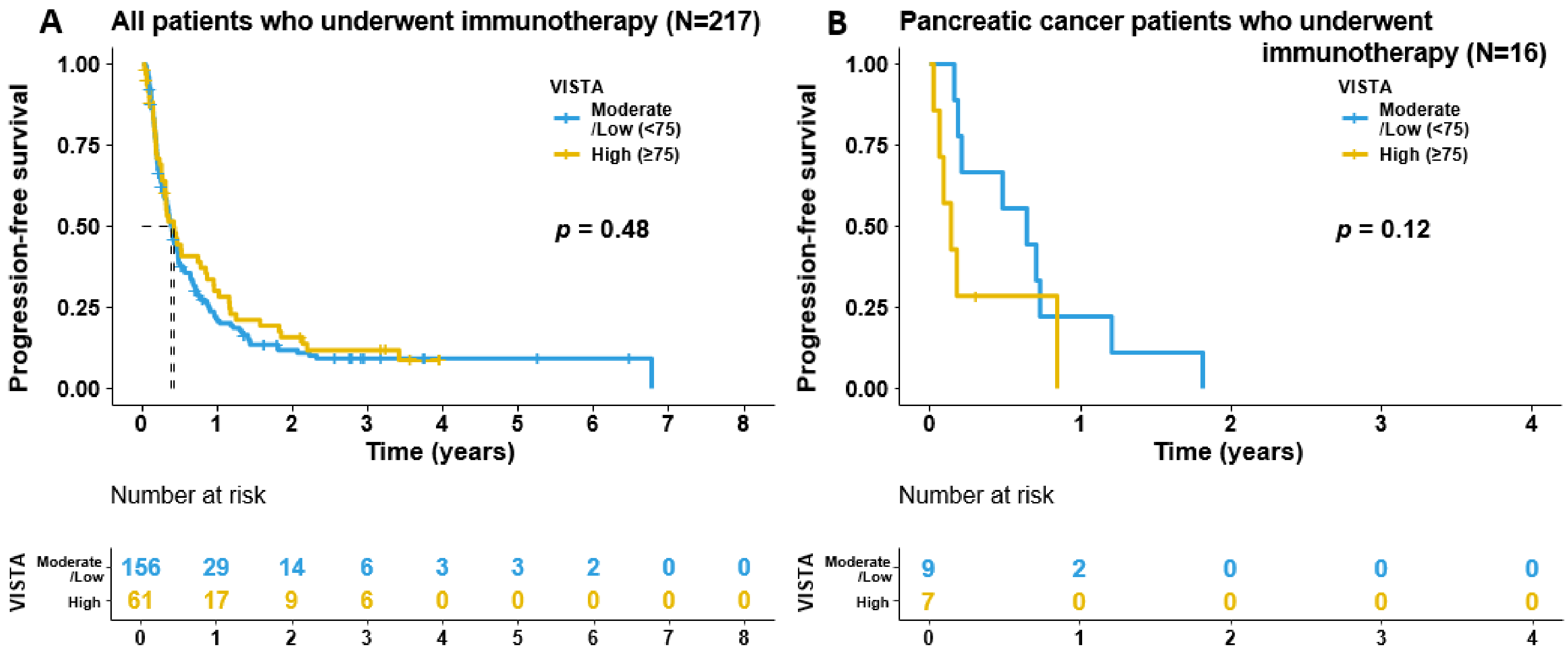

Definition of RNA expression: High = 75–100; Moderate/Low = 0–75 percentile rank value score.

Kaplan-Meier curves evaluate the duration from the date of immunotherapy initiation to the date of the earliest of disease progression (clinical or radiological) or death from any cause. **A**) Among all ICI-treated patients, the median progression-free survival after the initiation of immunotherapy was 0.42 years (95% CI, 0.31–0.86) for the high-VISTA group and 0.39 years (95% CI, 0.33–0.48) for the low-VISTA group (p = 0.48). **B**) Pancreatic cancer patients (N=16) had median PFS of 0.14 years for the high-VISTA group and 0.64 years for the low-VISTA group, respectively (p = 0.12).

Supplemental References

S1. Postow MA, Callahan MK and Wolchok JD. Immune Checkpoint Blockade in Cancer Therapy. Journal of Clinical Oncology: Official Journal of the American Society of Clinical Oncology. 2015;33(17):1974-82.

S2. Khunger M, Hernandez AV, Pasupuleti V, Rakshit S, Pennell NA, Stevenson J, et al. Programmed Cell Death 1 (PD-1) ligand (PD-L1) Expression in Solid Tumors As a Predictive Biomarker of Benefit From PD-1/PD-L1 Axis Inhibitors: A Systematic Review and Meta-Analysis. JCO precision oncology. 2017;1:1-15.

S3. Yearley JH, Gibson C, Yu N, Moon C, Murphy E, Juco J, et al. PD-L2 Expression in Human Tumors: Relevance to Anti-PD-1 Therapy in Cancer. Clinical Cancer Research: Official Journal of the American Association for Cancer Research. 2017;23(12):3158-67.

S4. Buchbinder EI, Desai A. CTLA-4 and PD-1 Pathways: Similarities, Differences, and Implications of Their Inhibition. Am J Clin Oncol. 2016;39(1):98-106.

S5. Rodriguez-Barbosa JI, Schneider P, Weigert A, Lee KM, Kim TJ, Perez-Simon JA, et al. HVEM, a co-signaling molecular switch, and its interactions with BTLA, CD160, and LIGHT. Cell Mol Immunol. 2019;16(7):679-82.

S6. Wolf Y, Anderson AC, Kuchroo VK, et al.. TIM3 acts as an inhibitory receptor. Nature reviews Immunology. 2020;20(3):173-85.

S7. Andrews LP, Cillo AR, Karapetyan L, Workman CJ, Vignali DAA. Molecular pathways and mechanisms of action of LAG3 in cancer therapy. Clinical Cancer Research: Official Journal of the American Association for Cancer Research. 2022;28(23):5030-9.

S8. Johnston RJ, Su LJ, Pinckney J, Critton D, Boyer E, Krishnakumar A, et al. VISTA is an acidic pH-selective ligand for PSGL-1. Nature. 2019;574(7779):565-70.
